# Comparison of quality of chest compressions during training of laypersons using Push Heart and Little Anne manikins using blinded CPRcards

**DOI:** 10.1186/s12245-017-0147-6

**Published:** 2017-06-24

**Authors:** Shota Tanaka, Alexander E. White, Ryo Sagisaka, Guanseng Chong, Eileen Ng, Jinny Seow, Nurul Asyikin MJ, Hideharu Tanaka, Marcus Eng Hock Ong

**Affiliations:** 1 Research Institute of Disaster management and EMS, Kokushikan University, Tokyo, Japan; 2 Unit for Pre-Hospital Emergency Care, Singapore General Hospital, Singapore, Singapore; 3 Department of EMS System, Graduate School, Kokushikan University, Tokyo, Japan; 4 Department of Emergency Medicine, Singapore General Hospital, Singapore, Singapore; 5 Health Services and Systems Research, Duke-NUS Medical School, Singapore, Singapore

**Keywords:** Chest compression, CPR quality, Push Heart, Feedback, CPRcard

## Abstract

**Background:**

Mass Cardio-Pulmonary Resuscitation (CPR) training using less expensive and easily portable manikins is one way to increase the number of trained laypeople in a short time. The easy-to-carry, low-cost CPR training model called Push Heart (PH) is widely used in Japan. The aim of this study was to examine if PH can achieve chest compression quality that is similar to that using more conventional Little Anne (LA) manikins for training laypersons.

**Methods:**

This prospective randomized crossover study was done during routine community CPR training of laypersons in Singapore. The participants were randomly allocated into two groups, using the PH and LA models respectively. They crossed over during the training so that both groups had measurements using both models. Chest compression data were collected using blinded CPRcards, which are credit card-sized devices with accelerometers and data capture. Participants did not receive any CPR feedback during measurement.

**Results:**

Forty-two people had data captured for the study with 15 males. The median compression depth was 41.5 mm on LA and 38.0 mm on PH (*p* = 0.0664), and median compression rate was 105 cpm on LA and 103 cpm on PH (*p* = 0.2429). Overall, only 1.5% of compressions performed on the PH achieved adequate depth of between 50–70 mm compared to 5.5% achieved on LA (*p* = 0.049). In contrast, 84% of all compressions performed on the PH were within the adequate rate of 100–120 cpm compared to 79.5% on LA (*p* = 0.457). Only the under 20-year-old group was able to achieve adequate median compression depth (50.5 mm) on LA, while the older age groups did not (*p* = 0.0024). The other age groups performed similar quality of chest compression regardless of the model used. 73.8% of participants preferred the LA for training. After the training, participants felt similarly well-prepared with either model with a median score of 8/10 on LA compared to 7/10 on PH (*p* = 0.0011).

**Conclusions:**

The PH can be an alternative mass CPR training model. Both models achieved satisfactory chest compression rates, but the majority of participants, especially the elderly, had difficulty achieving adequate depth.

## Background

Out-of-hospital cardiac arrest (OHCA) is a major cause of death in the world. Every year, emergency medical services (EMS)-assisted OHCA was experienced by 359,800 people in the USA [1]. According to a report in 2011, the survival to discharge rate was 31.4% for bystander-witnessed OHCA in the USA [1]. Without cardiopulmonary resuscitation (CPR) and defibrillation, the survival rate decreases from 7 to 10% every minute [2]. It takes about 8 min from activating EMS for an ambulance to arrive at the scene in many countries [3]. According to a report of the Japanese Fire and Disaster Management Agency, 8.6 min was the average response time in 2015 [4]. It has been shown that if CPR can be started by laypersons while waiting for ambulance arrival, the survival rate improves significantly [5]. Compression-only CPR is recommended for bystanders by the Japan Circulation Society and the American Heart Association (AHA) [6, 7].

Increasing the rate of bystander CPR is the goal of CPR training, which may eventually increase the OHCA survival rate [8]. Mass CPR training is an important way to achieve an optimal level of saturation of CPR trained residents, which may result in more lives saved [9]. While increasing the CPR training rates is prudent, it is important to be mindful of and address the barriers to performing CPR in an emergency in planning these initiatives [10, 11]. In Japan, mass CPR training and school CPR training have been increasing in popularity [12]. An easy-to-carry, low-cost CPR training model, called the Push Heart, has become popular for a mass CPR training in Japan.

The Little Anne is another commonly used CPR training manikin [13]. It is more life-like but is more costly to purchase and less convenient for mass CPR training. The logistic and labor involved in preparing for a mass training, particularly when training requires transporting many Little Annes for a mass CPR training. In comparison, the Push Heart made of a sponge-like material is light and easy to carry. One person can carry 50 Push Hearts at one time. The durability of the Push Heart is unknown, but one may surmise that it is less durable than the Little Anne which is made of heartier rubber-like material.

Little research has been conducted to compare the CPR quality between different types of CPR training models. Our study aimed to determine if Push Heart can achieve chest compression quality that is similar to that on Little Anne, and to conduct a simple survey afterwards on user experience and thoughts. The hypothesis of the study was that no difference in CPR quality between Push Heart and Little Anne would be found. We aimed to study if Push Heart can be a practical alternative to the Little Anne for CPR training of laypeople.

## Methods

The SingHealth Centralised Institutional Review Board approved this study under CIRB #2015/2475. A randomized cross-over design was used, and the data collection took place during a community-based DARE CPR + AED training session.

Participants’ performed two 2-min rounds of compressions; one round per CPR compression model being studied. There was a 3-min recovery time between cross-over. Primary outcome was the adequate depth and rate, as well as compression depth and rate. Secondary outcome was the scores from the survey.

### Participants

**Fig. 1:**
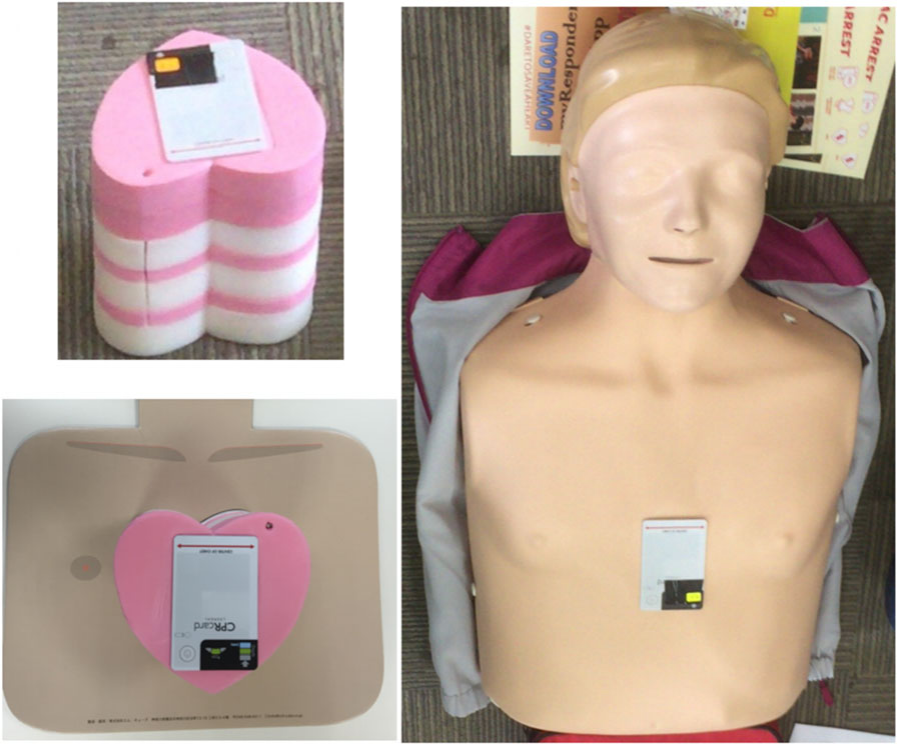
CPRcard on Push Heart (*left*) and CPRcard on Little Anne (*right*)

A total of 42 laypeople participated in this DARE CPR + AED training study (Fig. 1). Their ages ranged from <20 to over 70 years old, with a third between ages 51 and 60. Sixty-four percent were female. Most participants were above 160 cm in height (60%), and between 60 and 79 kg in weight (40%). The participants were randomly allocated between two groups; group A and group B.

### Instrumentation

Two chest compression training models were used in this study. The Push Heart (ぷっしゅハート®) is an easy-to-carry, low-cost CPR training model and is manufactured by M-Cube (Yokohama, Japan). The Little Anne™ is a CPR manikin that is manufactured by Laerdal (Stavenger, Norway) and is widely used globally for CPR + AED training. One Little Anne costs $239 USD and it weighs 3900 g [14]. On the other hand, one Push Heart costs $21.60 USD (or about 2160 Yen) [15] and it weighs 55 g. To record compression data consistently, CPRcards (manufactured by Laerdal Stavanger, Norway) were used (Fig. 1). CPRcards record data on rate and depth of compressions along with several other compression variables.

### Study procedure

**Fig. 2:**
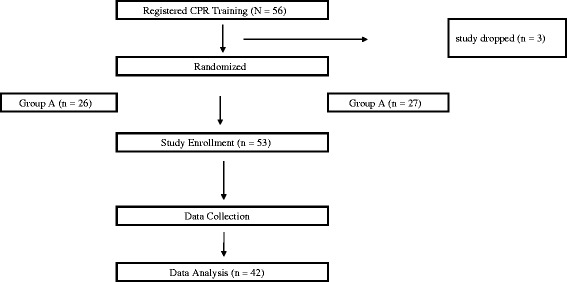
Flow chart for the procedure

The flow chart for the study is shown in Fig. 2. Prior to beginning the study session, participants were randomly assigned a seat. The seat number was allocated and noted on their consent form. Group A and group B were paired up and went through CPR training and compression data collection process. The data collection was included within the DARE CPR training. One pair of participants shared one Little Anne, a Push Heart, and an AED. Group A performed chest compression on Little Anne first and then Push Heart after. Group B performed chest compression on Push Heart first and then Little Anne after.

The DARE CPR training is a video-based instruction. DARE CPR skill training has three main parts, “Call 995,” “Push Hard, Push Fast,” and “AED”. During the training Group A performed 2 min of chest compression on Little Anne. Group B would perform 2 min of chest compression on Push Heart at the same time. In both cases blinded CPRcards were used to measure compression data. Subsequently groups A and B switched roles and performed compressions accordingly. A survey was administered after the session to attain the factors.

### Statistical analysis

All data were analyzed by JMP, v. 11.0 (the SAS Institute Inc.). Median and interquartile range were calculated for continuous or ordinal variables and compared by Wilcoxon signed-rank test or Kruskal-Wallis test. The total number and percentage were calculated for categorical variables and compared by Pearson’s chi-squared test. The relationship between the manikin and depth average was evaluated by scatterplot and calculating linear regression. Statistical analysis used a two-tailed hypothesis test, and the level of significance for decision-making was set at α = .05.

## Results

### Participant’s demographics

Forty-two laypersons with no medical training participated in the study. Study procedure flowchart is described in Fig. 1. As shown in Table 1, 33.3% were in the range of the 61–70-year-olds, 35.7% were male, 40.5% were in the range of the 160–169 cm in height, and 31.0% were in the weight range of 50–59 kg.

**Table 1:** The participants demographics

	*n* (%)
Gender	
Male	15 (35.7)
Female	27 (64.3)
Age (years)	
< 20	8 (19.0)
21–30	6 (14.3)
31–40	2 (4.8)
41–50	7 (16.7)
51–60	14 (33.3)
61–70	3 (7.1)
> 70	2 (4.8)
Height (cm)	
140–149	2 (4.8)
150–159	15 (35.7)
160–169	17 (40.5)
170–179	7 (16.7)
180–189	1 (2.4)
Weight (kg)	
< 40	1 (2.4)
40–49	9 (21.4)
50–59	13 (31.0)
60–69	12 (28.6)
70–79	5 (11.9)
80–89	1 (2.4)
90–99	0 (0.0)
> 99	1 (2.4)

Pearson’s chi-squared test

### Primary outcome: depth and rate

The median for the average percentage of the targeted depth achieved was 5.5% on Little Anne and 1.5% on Push Heart (*p* = 0.0498; Table 2). The median for the average depth was 41.5 mm on Little Anne and 38.0 mm on Push Heart (*p* = 0.0664; Table 2). The median for the average percentage of the targeted rate achieved was 79.5% on Little Anne and 84.0% on Push Heart (*p* = 0.4575; Table 2). The median average rate was 105.0 compressions per minute (cpm) on Little Anne and 103.0 cpm on Push Heart (*p* = 0.2429; Table 2).

**Table 2:** The primary outcome on Little Anne and Push Heart

	Little Anne	Push Heart	*p* value
Compression depth (mm)	41.5 (33.0–48.0)	38.0 (31.8–41.0)	0.0664
Compression rate (cpm)	105.0 (101.0–109.5)	103.0 (101.0–105.5)	0.2429
Adequate depth (%)	5.5 (0–42.5)	1.5 (0.0–7.5)	0.0498*
Adequate rate (%)	79.5 (69.0–90.3)	84.0 (67.3–93.3)	0.4575

Median(IQR), Wilcoxon test, two-tails

*p < 0.05 significant

As shown in Table 3, the median average depth on Push Heart was 39 mm performed by males and 38 mm performed by females (*p* = 0.6358). The median average depth on Little Anne was 44 mm performed by males and 38 mm performed by females (*p* = 0.0344; Table 3). A statistically significant difference was found between genders among the average depth on Little Anne. The median average depth achieved on Little Anne was 50.5 mm performed by the under 20-year-olds, 43.0 mm performed by the 21–50-year-olds, 33.0 mm performed by the 61–70-year-olds, and 39.5 mm performed by the over 70-year-olds (*p* = 0.00024; Table 2). A statistically significant difference was found between age groups. There were no significant differences in compression performance based on the weight and height.

**Table 3:** Baseline characteristics and CPR performance

	*n*	PH average depth (mm)	*p* value	PH adequate depth (%)	*p* value	LA average depth (mm)	*p* value	LA adequate depth (%)	*p* value
Gender									
Male	15	39.0 (30.0–41.0)	0.6358	2.0 (0.0–5.0)	0.8823	44.0 (42.0–48.0)	0.0344*	14.0 (3.0–47.0)	0.0611
Female	27	38.0 (32.0–42.0)		1.0 (0.0–9.0)		38.0 (30.0–48.0)		2.0 (0.0–41.0)	
Age group (year old)									
< 20	8	39.5 (33.0–41.5)	0.2644	3.5 (1.0–11.25)	0.1683	50.5 (42.75–54.75)	0.0024*	58.5 (22.25–88.75)	0.0004*
21–50	15	40.0 (33.0–47.0)		2.0 (0.0–26.0)		43.0 (39.0–46.0)		9.0 (3.0–26.0)	
61–70	17	35.0 (30.0–39.5)		0.0 (0.0–2.0)		33.0 (29.0–41.5)		0.0 (0.0–2.5)	
> 70	2	38.0 (24.0–52.0)		36.5 (0.0–73.0)		39.5 (24.0–55.0)		48.5 (80.0–97.09)	
Height (cm)									
140–149	2	40.0 (36.0–44.0)	0.3985	9.0 (1.0–17.0)	0.0607	42.0 (30.0–54.0)	0.3133	46.0 (0.0–92.0)	0.1795
150–159	15	35.0 (31.0–40.0)		0.0 (0.0–4.0)		39.0 (28.0–48.0)		0.0 (0.0–41.0)	
> 160	25	39.0 (31.0–41.5)		2.0 (0.5–10.0)		42.0 (37.5–47.0)		9.0 (2.0–36.5)	
Weight (kg)									
< 49	10	35.5 (31.75–40.0)	0.8051	2.0 (0.0–10.25)	0.6269	41.0 (31.5–53.25)	0.8325	15.0 (0.0–68.5)	0.6446
50–59	13	37.0 (31.0–43.0)		0.0 (0.0–14.5)		40.0 (30.5–43.5)		5.0 (0.0–11.5)	
60–79	17	39.0 (33.5–41.0)		2.0 (0.5–6.0)		42.0 (36.0–47.0)		3.0 (0.5–36.5)	
> 80	2	35.0 (30.0–40.0)		5.5 (2.0–9.0)		42.5 (28.0–75.0)		47.5 (1.0–94.0)	

Median(IQR), Wilcoxon test, two-tails

*p < 0.05 significant

### Secondary outcome: survey

Participants were administered a survey after completing the session. They were asked probed on such matters as: on which CPR training model they preferred to perform chest compressions (question 5); to rate if how much they liked each device (question 6); the most important characteristic for each CPR training model (question 7–8); and to rate how well they feel the training prepared them to perform CPR on each model (question 9).

Based on the results, 73.8% preferred Little Anne and 19.0% preferred Push Heart (Table 4). Regarding the most important characteristic for Little Anne, 45.2% chose “life-like/realistic” (Table 4). For Push Heart, 16.7% chose “more portable.” One participant provided a comment about the Push Heart writing that it was “unstable during compressions”.

**Table 4:** Initial survey questions and results

	*n* (%)
Preference	
Little Anne	31 (73.8)
Push Heart	8 (19.0)
No answer	1 (2.4)
The most important characteristic of the Little Anne	
1. Did not prefer	2 (4.8)
2. Ease of use	6 (4.8)
3. More durable	2 (4.8)
4. Life-like/realistic	19 (45.2)
5. Feels more like proper equipment	5 (11.9)
6. Other	0 (0)
The most important characteristic of the Push Heart	
1. Did not prefer	15 (35.7)
2. More portable	7 (16.7)
3. Shape and color	2 (4.8)
4. Ease of use	6 (14.3)
5. Less expensive	3 (7.1)
6. Other	1 (2.4)

Pearson’s chi-squared test

For question 6, participants were asked, “Please rate how much you liked each device on a scale of 1–10, where 1 means you do not like it at all, and 10 means you like it a lot.” The median score was 8 (25%: 8; 75%: 10) on Little Anne and 7 (25%: 6; 75%: 8.25) on Push Heart (*p* = 0.0017; Table 5). Question 9 asked, “Based on how you feel now after using the Little Anne and the Push Heart, please rate each on how well you feel the training prepared you to perform CPR (1 means you do not feel well prepared and 10 means you feel very well prepared that you can perform CPR)”. The median score was 8 (25%: 7; 75%: 9.25) on Little Anne and 7 (25%: 5; 75%: 8.25) on Push Heart (*p* = 0.0011; Table 5).

**Table 5:** Factors associated with the preference between Little Anne and Push Heart

	*n*	Median	25%	75%	*p* values
Question 6 “A Rate of how much participants liked”					
Little Anne	42	8	8	10	0.0017*
Push Heart	42	7	6	8.25	
Question 9 “A Rate of how much participants feel well prepared”					
Little Anne	42	8	7	9.25	0.0011*
Push Heart	42	7	5	8.25	

*p < 0.05 significant

Survey question (1 = you do not like it at all, 10 = you like it a lot)

## Discussion

In this study, we found participants performed higher adequate depth (percentage of total within proper range) on Little Anne, while there was no difference in the adequate rate. No difference was found in compression performance among baseline characteristics. Males compressed deeper on Little Anne, and the under 20-year-olds were the only group that compressed over 50 mm on Little Anne. According to the survey results, Little Anne was the preferred training model to use.

Adequate compression depth and percentage of adequate chest compression depth are typically used to compare quality in previous research [16]. We found the percentage of adequate depth was not ideal in this study, with a wide interquartile range. Statistically there were differences in the percentage of adequate depth achieved between Little Anne and Push Heart. The median depth was 41.5 mm on Little Anne and 38.0 mm on Push Heart.

In our study, the participants were not able to achieve good performance with regards to depth of compression. However, this is consistent with previous studies looking at CPR training in laypersons. In a previous study by Kramer-Johansen et al. (2006), the percentage of adequate depth performed by ambulance personnel was increased to 53% with a feedback device from 24% without a feedback device [17]. Therefore, training with a feedback device is the most effective method to teach high quality CPR [17–21].

Based on questions 6 and 9 from the survey, participants preferred Little Anne as a CPR training model and they feel better prepared to perform chest compressions after they practice on Little Anne. There were a few participants that preferred the Push Heart. We found the average depth achieved was similar for both models. The participant’s preference and confidence level in the Push Heart were slightly lower than that of Little Anne.

In this study of laypersons, we found the quality of chest compressions and especially depth was generally low. CPR training data is not easy to measure in large groups of participants, we found the CPRcard was an excellent device that can collect a large amount of training data at the same time. However, we note that the CPRcards were calibrated to 50–70 mm as the targeted depth. The 2015 AHA and Japan Resuscitation Council (JRC) guidelines are 50–60 mm as an appropriate depth [22, 23]. From our results, the average depth of 42 participants was around 40 mm regardless of the CPR training model. The percentage of adequate depth was not good throughout the compression period regardless of CPR training models.

Mass CPR training is the best way to quickly increase numbers of potential laymen first responders. Residences or homes are the place that 70% of OHCA cases occur [1]. CPR by laypersons is critical to save lives. Although our results suggest the Little Anne is the preferred CPR training model, the Push Heart could achieve about the same depth during training; and portability and lower cost could be a reason to use Push Heart for mass CPR training. Push Heart could be a good alternative for mass CPR training. Both models achieved high quality of chest compression rates. Difficulty in achieving adequate depth was seen in the elderly.

From our results, the average depth achieved was 41 mm, even on the Little Anne. That is the general average depth achieved by laypeople who were trained in CPR for the first time. The average compression depth for the highest survival rate was 45.6 mm in a real world study [24]. Participants needed to push harder to reach the depth correlating to the highest survival rate. A feedback device is a critical tool to perform adequate depth; and the CPRcard shows promise in this regard. Training with a feedback device and performing chest compressions with a feedback device in an emergency in the field are the best ways to teach and deliver high quality chest compressions.

## Conclusions

We found that the Push Heart can be an alternative mass CPR training model for laypersons. Both models achieved satisfactory chest compression rates, but the majority of participants, especially the elderly, had difficulty achieving adequate depth. We found the CPRcard to be a promising device to measure CPR quality during mass training.

## Abbreviations

AHA: American Heart Association
cpm: Compressions per minute
CPR: Cardiopulmonary Resuscitation
DARE: Dispatcher assisted first responder
EMS: Emergency medical services
JRC: Japan Resuscitation Council
LA: Little Anne
OHCA: Out-of-hospital cardiac arrest
PH: Push Heart
SGH: Singapore General Hospital
